# Supplementary material for: Reduced utilitarian willingness to violate personal rights during the COVID-19 pandemic
Source: PLoS One. 2021 Oct 22;16(10):e0259110. doi: 10.1371/journal.pone.0259110 (PMC8535394; doi:10.1371/journal.pone.0259110)
Supplement: S1 Appendix — Moral reasoning task with the follow up questions on confidence/emotionality and the control question, categorized by moral category: Non-Moral (NM), Impersonal Moral (IM), Personal Right (PR), Agent-centered Permissions (AP), and Special Obligations (SO). Per our pre-registered analysis, only responses to PR, AP and SO dilemmas are reported in this paper. (RTF) [file pone.0259110.s001.rtf]

S1 Appendix (Moral Reasoning Task)
Non-Moral category
Q11.1. You are at home one day when the mail arrives. You receive a letter from a company that provides financial services. You have heard of this company, which has a good reputation. They have invited you to invest in a mutual fund.  The minimum investment for this fund is $1000. You already know a lot about this particular mutual fund. It has performed poorly over the past few years. Based on what you know, there is no reason to think that it will perform any better in the future. Would you invest $1000 in this mutual fund in order to make money?
Q11.2. How confident are you that this was the right choice?
Q11.4. How emotional were you when thinking about the situation?
Q11.5. Do you expect this investment to make a lot of money?

Q19.1. You need to travel to a nearby city in order to attend a meeting that starts at 2:00 PM. You can either take the train or the bus. The train will get you there just in time for your meeting no matter what. The bus is scheduled to arrive an hour before your meeting. However, the bus sometimes is several hours late because of traffic. It would be nice to have an extra hour before the meeting, but it is very important that you arrive on time. Would you take the train instead of the bus in order to ensure that you are not late for your meeting?
Q19.2. How confident are you that this was the right choice?
Q19.4. How emotional were you when thinking about the situation?
Q19.5. Are you more likely to miss the meeting if you take the bus?

Q25.1. You are bringing home some plants from the store. You have lined the trunk of your car with plastic to catch the mud from the plants, but your trunk will not hold all of the plants you have bought. You could bring all of the plants home in one trip, but you would need to put some of the plants in the back seat. If you put the plants in the back seat, the mud from the plants will ruin your fine leather upholstery, which would cost thousands of dollars to replace.  Would you make two trips home to avoid ruining the upholstery of your car?
Q25.2. How confident are you that this was the right choice?
Q25.4. How emotional were you when thinking about the situation?
Q25.5. If you had made a single trip, would all the plants have fit in your trunk?

Q28.1. You have a very bad headache. You go to the pharmacy looking for your favorite brand of headache medicine. When you get there, you find that the pharmacy is out of the brand that you are looking for.  You have known the pharmacist at this store for a long time, and you trust him. He says he has a generic medicine that is “exactly the same” as the name-brand medicine that you wanted. In the past, he has always given you good advice. Would you keep looking for the name-brand medicine you came for, instead of buying the generic medicine?
Q28.2. How confident are you that this was the right choice?
Q28.4. How emotional were you when thinking about the situation?
Q28.5. Does the pharmacist think the generic medicine is just as good?

Impersonal Moral category

Q4.1. You work for the Government Health Agency. You must decide whether to promote a new vaccine. This vaccine will protect almost everyone who takes it from a deadly disease. However, the vaccine also carries a risk. A very small number of healthy people who take it will get the disease from the vaccine. You have carefully studied the safety of the vaccine. The chance that someone will die because they did not take the vaccine is much greater than the chance that they will die from the vaccine. Would you tell people to use this vaccine to prevent the disease?
Q4.2. How confident are you that this was the right choice?
Q4.4. How emotional were you when thinking about the situation?
Q4.5. Will fewer people die if the vaccine is widely used?

Q7.1. You are the driver of a runaway trolley approaching a fork in the tracks. On the tracks going to the left is a group of five railway workers. On the tracks going to the right is a single railway worker. If you do nothing, the trolley will go to the left, causing the five workers to die. The only way to avoid the deaths of these five workers is to hit a switch on your dashboard that will make the trolley go to the right, leading to the death of the single worker. Would you hit the switch to avoid the deaths of the five workers?
Q7.2. How confident are you that this was the right choice?
Q7.4. How emotional were you when thinking about the situation?
Q7.5. Will more people die if you hit the switch than if you donation?

Q16.1. You are on the board of a charity in a poor country. The main river there floods every year. Each time this happens, 5,000 people who live downstream are left homeless. These floods do not affect people who live upstream.  Some people in your charity want to build a dam to prevent these floods. This dam would cover a valley upstream with water. Villagers from nearby bring their cows to this valley to eat the grass. If this valley is covered with water, 1,000 of these villagers would have to move to find a new place to feed their cows.  Would you vote against building the dam so these villagers can stay where they are?
Q16.2. How confident are you that this was the right choice?
Q16.4. How emotional were you when thinking about the situation?
Q16.5. Will more people be displaced by the dam than would be displaced by the floods?

Q31.1. You are the night watchman in a hospital. One night, an accident in the building next door makes deadly chemicals enter the hospital's air ducts. If you don't do anything, these fumes will enter a room with three patients in it, and they will all die. The only way to save these three patients from dying is to hit a certain switch. This will keep the fumes out of the room with the three patients in it. Instead, the fumes will enter a room with a single patient in it, and he will die. Would you allow the fumes to enter the room with three patients so that the single patient will live?
Q31.2. How confident are you that this was the right choice?
Q31.4. How emotional were you when thinking about the situation?
Q31.5. Will more people die if you hit the switch than if you don't?

Personal Rights category	

Q3.1. “You are on good terms with your two neighbors, Jeffrey and Benjamin. Unfortunately, they are both hot-headed and hate each other. The other day, you saw Benjamin carelessly drop some trash near Jeffrey's yard.  Jeffrey knocks on your door, visibly angry, and asks if it was Benjamin who left the trash. If you tell him the truth, you know he will go to Benjamin's house and they will have a violent fight. If you lie and tell him that it was you who accidentally dropped the trash, Jeffrey will soon forget about the whole thing. Would you tell the truth that it was Benjamin who left the trash near Jeffrey's yard?
Q3.2. How confident are you that this was the right choice?
Q3.4. How emotional were you when thinking about the situation?
Q3.5. If you lie and say that it was you who dropped the trash, will Jeffrey get angry at you?

Q10.1. You are leading a rescue team for seven miners that are stuck in an underground mine, which is flooding. Six miners are trapped at the bottom and will drown if not rescued soon. One miner is trapped higher in the elevator shaft and will not drown. The only way to rescue the six at the bottom is to quickly send down the mine elevator. If you do this, the one miner in the shaft will be crushed to death. If you do not send down the elevator, you will have enough time to rescue the one miner in the shaft. Would you send down the mine elevator to rescue the six miners at the bottom?
Q10.2. How confident are you that this was the right choice?
Q10.4. How emotional were you when thinking about the situation?
Q10.5. If you do not send down the elevator, will the six miners live?

Q15.1. You are negotiating with a powerful and determined terrorist. He is about to set off a bomb that will kill thousands of people. Your one advantage is that you have his teen-age son under your control. There is only one thing you can do to stop him from setting off his bomb. You can contact him over the video connection that he has created and break one of his son's arms. You can then threaten to break the other one if he does not give himself up. Would you refrain from breaking the boy's arm even though the terrorist will kill thousands of people with his bomb?
Q15.2. How confident are you that this was the right choice?
Q15.4. How emotional were you when thinking about the situation?
Q15.5. Will more people die if you break the boy's arm?

Q18.1. You are a waiter. One of the regular customers of your restaurant will go to jail in 48 hours. You overhear him say that he plans to infect as many people as possible with HIV before he goes to jail. You know that he is telling the truth and has access to many potential victims. You also know that he has a strong allergy to poppy seeds. If he eats even one he will go into convulsions and have to be hospitalized for at least 48 hours. Would you cause this man to have a serious allergy attack to prevent him from spreading HIV?
Q18.2. How confident are you that this was the right choice?
Q18.4. How emotional were you when thinking about the situation?
Q18.5. If you do not cause this man to have an allergy attack, will more people get sick?

Q21.1. An epidemic has spread worldwide killing millions of people. You have developed two substances in your underground shelter. One of them is a cure but the other one is deadly. You don't know which is which.  Two people have run downstairs to your shelter trying to avoid the epidemic. The only way to identify the cure is to inject each of these people with one of the two substances. One person will live but the other will die. Then you will be able to start saving lives with the cure. Would you kill one of these people with a deadly injection to identify a cure that will save millions of lives?
Q21.2. How confident are you that this was the right choice?
Q21.4. How emotional were you when thinking about the situation?
Q21.5. Will more people live if you inject these two people with the unknown substances?

Q23.1. A runaway trolley is heading down the tracks toward five workers, and will kill them if it keeps going. You are on a footbridge over the tracks, in between the approaching trolley and the five workers. Next to you on this footbridge is a stranger who is very large. The only way to save the lives of the five workers is to push this stranger off the bridge and onto the tracks below where his large body will stop the trolley. The stranger will die if you do this, but the five workers will be saved. Would you push the stranger onto the tracks to save the five workers?	
Q23.2. How confident are you that this was the right choice?
Q23.4. How emotional were you when thinking about the situation?
Q23.5. Will more people die if you push the stranger than if you don't?

Q26.1. You are on a cruise ship when a fire breaks out, forcing everyone to abandon ship. The lifeboats are carrying many more people than they should. The seas start to get rough, and your lifeboat begins to fill with water. If you do nothing, the boat will sink before help arrives and everyone on board will die. However, there is an injured person on board who will not survive even if help comes. If you throw that person overboard the boat will stay afloat and the rest of the passengers will be saved. Would you keep this injured person on the boat instead of throwing him overboard?
Q26.2. How confident are you that this was the right choice?
Q26.4. How emotional were you when thinking about the situation?
Q26.5. If the injured person is kept on the boat, will he live?

Q29.1. You are leading a group that is lost in the wilderness. Your group includes a family of six with a genetic vitamin deficiency. A few people's kidneys contain large amounts of this vitamin. There is one such person in your group. The only way to save the lives of the six family members is to remove one of this man's kidneys and take the necessary vitamins from it. He will not die if you do this, but his health will get worse. He does not want to give his kidney, but you have the power to do what you choose. Would you allow this man to keep his kidney rather than save the vitamin-deficient family?
Q29.2. How confident are you that this was the right choice?
Q29.4. How emotional were you when thinking about the situation?
Q29.5. Will more people live if you take the man's kidney?

Agent-centered Permissions	 category

Q6.1. You have a very rare type of blood. There are some people who can only receive blood transfusions from you, though you cannot receive blood from them. One of these people is a stranger who is very sick and will require many blood transfusions in order to survive. To donate all the blood that this person will need to get well, you will have to live in the hospital for six months.  During this time you will undergo multiple large blood donations.  These donations will make you feel weak and tired most of the time. Would you agree to these repeated blood donations to keep this person from dying?
Q6.2. How confident are you that this was the right choice?
Q6.4. How emotional were you when thinking about the situation?
Q6.5. Will this person get better without your blood donations?

Q8.1. You are a poor farmer during an unusually severe drought. Fortunately, there is a spring on your farm with enough water for all your crops this year. The nearest farm is miles away, and that farmer will have to abandon his farm and move away because he has no water. You could give him some water for enough crops to keep his farm, but then you could only plant some of your own crops. Your farm would survive but would not make enough money to afford some improvements that you have planned. He will not be able to repay you. Would you keep your water so you can afford the improvements that you have planned?
Q8.2. How confident are you that this was the right choice?
Q8.4. How emotional were you when thinking about the situation?
Q8.5. Will the other farmer keep his farm if you do not help him?

Q13.1. You are a college student who is very good at science. During your studies you find that you do not enjoy doing research. You also discover that you are very interested in dance. You meet with a career advisor who has known many other students with the same skills and interests. She tells you that if you become a scientist, you will make discoveries that save many lives, but you will always feel unfulfilled. If you become a dancer, you will have a satisfying career but will not help many other people. Would you decide to become a scientist so that you can help more people?
Q13.2. How confident are you that this was the right choice?
Q13.4. How emotional were you when thinking about the situation?
Q13.5. Would you find a career as a scientist fulfilling?

Q17.1. Your favorite band will perform one final concert before retirement. You have never had the chance to see them perform, and you really want to go. You have reserved tickets for this concert for $150. One week before the concert, an earthquake in a faraway country leaves thousands of people homeless and without enough food. You could give up your concert tickets and donate the $150 to earthquake relief. This money would provide two families with food and shelter through the winter. Otherwise they will be cold and hungry but will survive. Would you keep your tickets so that you can see this final concert?
Q17.2. How confident are you that this was the right choice?
Q17.4. How emotional were you when thinking about the situation?
Q17.5. Would anyone's life be improved if you miss the concert?

Q22.1. On vacation, your cruise ship stops at an island, and you bring a few dollars with you onshore. You find some local fruit for sale, which you have heard about and are curious to try. Another passenger forgot to bring her money from the ship, and she finds a type of shell for sale that is only found on this island. She asks to borrow your money to buy a shell for her friend, who is a biologist and would treasure it. If you agree, she will pay you back on the ship but you will miss your chance to try the fruit. Would you keep your money so that you can try the fruit?
Q22.2. How confident are you that this was the right choice?
Q22.4. How emotional were you when thinking about the situation?
Q22.5. Can you buy the fruit and also lend this woman money to buy the shell?

Q27.1. While waiting for a bus you begin to talk with a stranger. You remember that you have seen him on TV news shows, and that he is a very rich but strange man. He says that he has $10,000 in his wallet, and then gives you a choice.  He says you can take the money for personal use. However, if you do not take the money, he will donate $1,000,000 to a charity to cure cancer. He will not make that donation if you take his money now. Given what you know about him, you are sure that he will keep his word.  Would you take the $10,000 for your own personal use?
Q27.2. How confident are you that this was the right choice?
Q27.4. How emotional were you when thinking about the situation?
Q27.5. Will the rich man make a larger donation if you do not take his money now?

Q32.1. You are at home one day when the mail arrives. You receive a letter from a charity. You have heard of this charity, which has won many awards for its work. The letter asks you to make a donation of $200.  The letter explains that this money will allow them to provide needed medical care to some poor people in another part of the world. If you do not make this donation, they will not be able to help as many people as they could if you do donate this money. Would you make this donation rather than keep the money for your own personal use?
Q32.2. How confident are you that this was the right choice?
Q32.4. How emotional were you when thinking about the situation?
Q32.5. Would anyone's life be improved by your donation?

Q34.1. You are on a flight arriving at a small airport at night, and are very tired. You stand in line and get the last taxi, as all the other drivers have gone home. Just then, another passenger runs up and asks to share a ride. If he rides with you, the driver will have to take a longer route and it will take you 20 more minutes to get to your destination. If he cannot ride with you, it will take him an hour longer to get to his hotel by bus. Would you let him share a taxi ride with you so that he will not spend another hour on the bus?
Q34.2. How confident are you that this was the right choice?
Q34.4. How emotional were you when thinking about the situation?
Q35.5. If you share the taxi ride, will you arrive at your destination faster?

Special Obligations category

Q5.1. You are camping with your own small child and another child. Both children eat leaves from a poisonous plant. If untreated, your child will have painful boils all over his body for a year. The other child ate more leaves, and he will have painful boils for three years if he does not get treatment. Neither will have permanent scars or damage. You have a medicine that will keep these boils from appearing if a child drinks it immediately. Unfortunately, you only have one dose of this medicine. The medicine will not work if the dose is shared between the children. Would you give the medicine to your own child rather than the other child?
Q5.2. How confident are you that this was the right choice?
Q5.4. How emotional were you when thinking about the situation?
Q5.5. If both children were not treated, would the other child suffer more than your own child would?

Q9.1. You are driving a motorboat when a cruise ship nearby begins to sink. Looking left, you see that your nephew, who was on the ship, is holding on to a piece of floating wood. If you drive towards him at full speed you can save him right before he sinks.  In the opposite direction is another piece of wood with four strangers clinging to it. By driving at full speed in the opposite direction, you have just enough time to save them. You only have enough time to rescue either your nephew or the four strangers. There are no other boats in the area. Would you save the four strangers instead of your nephew? 
Q9.2. How confident are you that this was the right choice?
Q9.4. How emotional were you when thinking about the situation?
Q.9.5. Is there enough time to save all four strangers and your nephew?

Q12.1. You are a doctor whose patient is on dialysis and is waiting for a kidney transplant. There is a new kidney that can be given either to your patient or another person. You have not met this other person, who is a little healthier than your patient. Based on your research, you know that the kidney would allow your patient to avoid dialysis for six more years. The kidney would allow the other person to avoid dialysis for nine more years. You can decide who will receive the kidney. Would you give the kidney to your own patient rather than the other person?
Q12.2. How confident are you that this was the right choice?
Q12.4. How emotional were you when thinking about the situation?
Q12.5. Other things being equal, will this kidney do more good for your patient than for the other person?

Q14.1. You are a lawyer whose client has pleaded guilty to a serious crime, and will go to jail for 20 years. You discover that the police have overlooked evidence that your client is also guilty of another crime. An innocent man will be jailed for this other crime for 20 years. You could suggest to the police that they look at this evidence again. You can do this in a subtle way so they think this is their idea and not yours. If they do, the innocent man will go free, but 15 years will be added to your client's sentence. Would you keep quiet about the evidence out of loyalty to your client?
Q14.2. How confident are you that this was the right choice?
Q14.4. How emotional were you when thinking about the situation?
Q14.5. If you keep quiet, will the innocent man spend as much time in jail as your client?

Q20.1. You are a parent whose young daughter plays soccer on a local team. You have been invited to have her join a team in a special regional league with better players and coaches. You think that she will learn more and have more fun on the regional team. However, she cannot stay on the local team and play for the regional team. If she leaves the local team, they will not have enough players and will not be able to play together. If she stays, both the local and regional teams will still have enough players. Would you keep your daughter on the local team so that all the children can keep playing?
Q20.2. How confident are you that this was the right choice?
Q20.4. How emotional were you when thinking about the situation?
Q20.5. Will it be better overall for the other children if your daughter stays on the local team?

Q24.1. You are a doctor researching a bad infection for which there is no cure. You discover a mold that makes a medicine that cures this infection. Before you can tell others about your results, your own patient contracts the infection and will die without treatment. In order to save her you must use up all of your mold. It would take two years to grow another batch. During this time, fifty other people will die of this infection. These people could be saved if you do not give the medicine to your patient and continue your research. Would you allow this patient to die in order to save many more lives in the future?
Q24.2. How confident are you that this was the right choice?
Q24.4. How emotional were you when thinking about the situation?
Q24.5. Will more people live if you save your own patient?

Q30.1. You have been put in charge of a new charity to find housing for homeless children. You can either focus on helping children in your own neighborhood or in other countries. Your budget has enough money to give seven children in your neighborhood a home, food, and clothes for one year. You pass these children on the streets every day. Because things are cheaper in other countries, the same amount could give fifty children there a home, food, and clothes. You will never meet these children. Would you help the fifty children in other countries, rather than the seven children in your own town?
Q30.2. How confident are you that this was the right choice?
Q30.4. How emotional were you when thinking about the situation?
Q30.5. Would you help more children overall by focusing on children in other countries?

Q33.1. Your best friend enjoys his work, though you know that he would still prefer to work for Company X. You have also heard that one of his coworkers recently lost her job and has been struggling to provide for her family. They both have similar skills and experience. You happen to meet the owner of Company X, and she mentions that she is planning to hire someone. Both your friend and his former coworker would be a good fit for this new job. You have an opportunity to suggest hiring either your friend or his former coworker. Would you suggest hiring your friend instead of his former coworker for this job?
Q33.2. How confident are you that this was the right choice?
Q33.4. How emotional were you when thinking about the situation?
Q33.5. Would it be more important for your friend to get this job than for his former coworker?
