# Supplementary material for: Reduced utilitarian willingness to violate personal rights during the COVID-19 pandemic
Source: PLoS One. 2021 Oct 22;16(10):e0259110. doi: 10.1371/journal.pone.0259110 (PMC8535394; doi:10.1371/journal.pone.0259110)
Supplement: S1 Table — Descriptive statistics for the actions adopted are represented as count (percentages) for participants who met the inclusion criteria in 2020 wave (N = 133). (DOCX) [file pone.0259110.s002.docx]

**S2 Table**. **Descriptive statistics: Actions adopted against COVID-19**

| **Actions** | **Response** | **2020 wave** |
| --- | --- | --- |
| **Wear Mask** |  |  |
|  | Yes | 130 (97.7%) |
|  | No | 3 (2.3%) |
|  | (Missing) | 0 (0.0%) |
| **Stayed Home** |  |  |
|  | Yes | 127 (95.5%) |
|  | No | 2 (1.5%) |
|  | (Missing) | 4 (3.0%) |
| **Travel Plans** |  |  |
|  | Yes | 116 (87.2%) |
|  | No | 7 (5.3%) |
|  | (Missing) | 10 (7.5%) |
| **Avoid Gatherings** |  |  |
|  | Yes | 107 (80.5%) |
|  | No | 8 (6.0%) |
|  | (Missing) | 18 (13.5%) |
| **Emergency Storage** |  |  |
|  | Yes | 99 (74.4%) |
|  | No | 33 (24.8%) |
|  | (Missing) | 1 (0.8%) |

*Note:* Descriptive statistics for the actions adopted are represented as count (percentages) for participants who met the inclusion criteria in 2020 wave (N = 133).
