# Supplementary material for: Reduced utilitarian willingness to violate personal rights during the COVID-19 pandemic
Source: PLoS One. 2021 Oct 22;16(10):e0259110. doi: 10.1371/journal.pone.0259110 (PMC8535394; doi:10.1371/journal.pone.0259110)
Supplement: S4 Table — The coefficient Estimate contains the intercept in the first row and the slopes (beta) at the following ones. SE represents the standard error, LL and UL the lower and upper limits of the confidence interval, t the t test statistic and p the probability value (N = 242). (DOCX) [file pone.0259110.s005.docx]

**S5 Table.** **Self-reported emotionality – personal rights dilemmas**

| **Term** | ***Estimate*** | ***SE*** | **95% CI** | | ***t*** | ***p*** |
| --- | --- | --- | --- | --- | --- | --- |
|  |  |  | ***LL*** | ***UL*** |  |  |
| **Intercept** | 2.61 | 0.10 | 2.40 | 2.81 | 24.86 | < .001 |
| **Wave 2** | 0.03 | 0.12 | -0.22 | 0.27 | 0.23 | .82 |
| **Age (years)** | 0.00 | 0.01 | -0.02 | 0.02 | 0.36 | .72 |
| **Education (years)** | -0.02 | 0.03 | -0.08 | 0.04 | -0.64 | .53 |
| **Female Gender** | 0.48 | 0.13 | 0.23 | 0.72 | 3.77 | < .001 |

*Note:* The coefficient *Estimate* contains the intercept in the first row and the slopes (beta) at the following ones. *SE* represents the standard error, *LL* and *UL* the lower and upper limits of the confidence interval, *t* the t test statistic and *p* the probability value (*N* = 242).
