# Supplementary material for: Reduced utilitarian willingness to violate personal rights during the COVID-19 pandemic
Source: PLoS One. 2021 Oct 22;16(10):e0259110. doi: 10.1371/journal.pone.0259110 (PMC8535394; doi:10.1371/journal.pone.0259110)
Supplement: Attachment — Submitted filename: Response to Reviewers.docx [file pone.0259110.s007.docx]

Dear Dr. Valerio Capraro,

Thank you for giving us the opportunity to submit a revised draft of the manuscript titled “No utilitarians in a pandemic? Shifts in moral reasoning during the COVID-19 global health crisis” to *PLOS ONE*. We appreciate the time and effort that you and the reviewers have dedicated to providing your valuable feedback on this manuscript. We are grateful to the reviewers for their insightful comments on the paper. We have been able to incorporate major changes to reflect the suggestions provided by the reviewers. Below you may find a point-by-point response to the reviewers’ comments and concerns.

**Comments from Reviewer 1**

Comment 1: *An important weakness of the research is the absence of a true control group (old people with no fear of the COVID-19).* *The participants were drawn from the part of the Hillblom Aging Network, and I’m left puzzled why the authors did not try to collect additional data selecting participants from the network who did not exhibit anxiety signs regarding COVID-19.*

Response 1: Thank you for this suggestion. In the preregistration (https://osf.io/g2wtp), for this reason we had proposed conducting a sensitivity analysis only including wave 2 participants who either (1) described themselves as either very worried or somewhat worried about the coronavirus outbreak, or (2) have taken at least two of the five described possible responses to the coronavirus outbreak. However, all respondents in wave 2 met criterion (2) of having taken at least two of the five possible responses to the pandemic, so this analysis would have been superfluous; more broadly, we conclude that all of the participants in wave 2 were practically affected by concerns about the pandemic. For this reason, the primary comparison was between the wave 1 cohort prior to the pandemic and the wave 2 cohort during the pandemic, although this does not exclude all other potential differences between cohorts. (At the same time, we note that a selected subgroup of older adults during COVID-19 that were unafraid of COVID-19 would itself have unique features distinguishing it from the wave 1 cohort, such that this might itself have introduced other uncontrolled differences in risk tolerance or anxiety.) To address the reviewer’s concerns given the patterns of response in our cohorts, we have conducted the following:

- While all of our wave 2 respondents met our prespecified criterion for being affected by the pandemic, we have conducted a further analysis to examine how different degrees of anxiety affect utilitarian responses (see “Result” section “Utilitarian judgments - overall and in personal rights dilemmas” lines 305-313). For this we split the sample of the 2020 wave in two categories: Worried (by collapsing the levels: Very worried and Somewhat worried) and Not worried (by collapsing the levels: Not too worried and Not at all worried). Estimated coefficients and p values differed only trivially with no significant effect of low level of worry on utilitarian responses.
- Emphasized this point and the asymmetry of our sample in our discussion (see “Discussion” section lines 368-372). Most participants in the 2020 wave had changed their lives in at least two ways in response to the pandemic (reporting worry and adopting a measure), which naturally evokes an asymmetry between the two samples.

Comment 2: *Regarding the context, much more could be said about moral utilitarianism during the COVID-19 pandemic.* *The results presented in this paper are interesting: old people are less utilitarian in the context of COVID-10 pandemic. Yet this is something quite opposed to what happens worldwide:* *health care provided have come in several states to endorse utilitarianism because of a lack of resources (for instance, by favoring the young at the expense of the older). Although I’m aware that this is not the very same topic, some discussion might be interesting and might help improve the discussion section.*

Response 2: We agree with this suggestion and we have, accordingly, revised the discussion to emphasize this point (see “Discussion” section lines 420-430). We added a paragraph combining our findings and the points raised in the proposed reference: “Vearrier, L., & Henderson, C. M. (2021, June). Utilitarian principlism as a framework for crisis healthcare ethics. In *Hec Forum* (Vol. 33, No. 1, pp. 45-60). Springer Netherlands.”

Comment 3: *Finally, what is the take-home message of your research? Why is it an important one?* *The discussion section should answer more thoroughly such questionings.*

Response 3: We agree with this and we have included your suggestion throughout the in the discussion section. We elaborated the discussion by emphasizing why this research is important and which the associated implications are (see “Discussion” section lines 431-442).

Comment 4: *Unless this is a requirement from the journal, please replace “Material and method” by Method. Material is a subsection of Method.*

Response 4: According to the journal’s requirements this element (Material and Method) can be renamed as needed. We have thus incorporated your suggestion throughout the manuscript (see “Method” section line 167).

Comment 5: *Please format all the Tables according to the APA7 guidelines*

Response 5: Thank you so much for your suggestion. We have followed the journal’s guidelines for tables (<https://journals.plos.org/plosone/s/tables> and https://journals.plos.org/plosone/s/file?id=wjVg/PLOSOne_formatting_sample_main_body.pdf), which depart slightly from the APA7 guidelines. (For example, the journal’s guidelines call for the title to be on the same line as the table number, and also display borders and lines separating each cell.)

**Comments from Reviewer 2**

Comment 1: *Several parts of the paper suggest that the authors are interpreting these findings in a causal way.* *Abstract: “We investigated the influence of…” (line 30) or Discussion: “we examined the effect of a global health crisis on…” (line 327). This study is 100% observational and the authors need to be clear and upfront about this.* *Moreover, they should avoid using terms such as “influence” or “effect” of the pandemic on moral choices given that the pandemic is not an experimental treatment. In one paragraph in the Discussion (lines 369-381), the authors acknowledge that many things happened between 2014 and 2020 and that the effect cannot be attributed to only the pandemic.* *Even if the results would have shown a clear and strong change in moral responses (which is not the case), this study cannot speak about the causal effect of the pandemic on moral choices (after all, it would be impossible to have such experimental treatment and the observational data presented here is clearly insufficient to suggest a causal role played by the pandemic).* *Similarly, this implies that this study cannot be thought of as a “real-world replication of the experimental manipulation in Tremoliere and colleagues” (line 339) since there is no experimental manipulation here.*
Response 1: Thank you for pointing this out. We agree with this comment. Therefore, we have changed the causal tone (by replacing words like effect or influence) with words that reflect better the observational nature of our study (e.g., context):

- in the “Abstract” (see lines 29-31): “We investigated moral judgments in the context of the contemporary global crisis among older adults, who are at greatest personal risk from the pandemic.”
- in the “Hypotheses and research questions” (see lines 143-144): “The current study utilizes a unique opportunity to study utilitarian judgments within the context of COVID-19 pandemic.”
- in the “Discussion” (see lines 336-338, 428-430, 431-434): e.g., “In this preregistered study, we examined individual judgments about hypothetical moral dilemmas during a global health crisis in a cohort of older adults at increased personal risk of severe complications from infection.” & “Non-utilitarian and utilitarian considerations may engage distinct social and individual ramifications depending on actors’ roles in a health-crisis context.” & “In summary, the present study represents a unique opportunity to examine utilitarian judgments during a global health crisis. Respondents during the COVID-19 pandemic gave fewer utilitarian responses to hypothetical dilemmas concerning conflicts between individual personal rights and the best overall outcome.”

We have accordingly removed our suggestion that this study can be thought of as a “real-world replication of the experimental manipulation in Tremoliere and colleagues” (see lines 347-348)

Comment 2: *The main result inspiring the title and the take-home message of the paper is based on one of the three categories of moral dilemmas (PRs). The authors claim to have planned and pre-registered “subsidiary analyses focusing exclusively on the subset of dilemmas involving personal rights” (line 159). However, the registry in the Open Science Framework (*[*https://osf.io/g2wtp*](https://urldefense.com/v3/__https:/osf.io/g2wtp__;!!LQC6Cpwp!-XS053PbuNseNc3dv_NX_8wOd21a8TdDhQCYb4xauwdWcbth53fXYPH6TiJO5TAwvfA$)*) does not support that claim. There is no mention to such subsidiary analysis which suggests that the focus on dilemmas involving PRs was indeed exploratory. Therefore, the significant result found using PR dilemmas should be labeled in that way. There is nothing inherently wrong with the data not supporting the main hypothesis and to try publishing the results of a subsequent exploratory analysis, but the authors should be explicit about it.*

Response 2: We appreciate this comment; the analysis restricted to PR dilemmas is labeled a “sensitivity analysis” in the preregistration (second to last paragraph): “we will repeat the primary analyses for hypotheses 1-3, but restricted to responses to PR dilemmas (which have been the main focus of dual process moral theories to date).” For this reason, we have first reported the primary analysis including dilemmas from all categories, before reporting the PR results. The PR-restricted analyses are planned and not *post hoc*.

Comment 3: *The title is misleading and problematic*.

Response 3: We agree, and we changed the title accordingly to reflect better the take home message of our study: “Reduced Utilitarian Willingness to Violate Personal Rights during the COVID-19 Pandemic”. See lines 3-4. We additionally have changed the subtitle of the study to: “Utilitarian Judgements during COVID-19” (see heading throughout the manuscript).

Comment 4: *As the authors correctly pointed out in lines 102-106, people might make utilitarian decisions for a variety of reasons that may or may not include engaging in utilitarian reasoning. In fact, DP theories (lines 107-126) suggest that making non-utilitarian decisions is consistent with a process different than reasoning, namely, emotional harm avoidance. So, one should not assume that the mechanism underlying the reduction in utilitarian choices is a “shift in moral reasoning” unless the data is accompanied by observations that support such mechanistic interpretation. Instead of presenting more data to test this mechanism, the authors could simply use mechanism-neutral language and refer to utilitarian “judgements” or “decisions”.*

Response 4: Thank you for pointing this out. We have accordingly replaced the moral reasoning term to utilitarian judgments in:

- subheadings of the “Introduction” (see line 84)
- the section “Hypotheses and research questions” (see lines 143-144)
- in the “Discussion” (see lines 431-432)

Comment 5: *This work studies moral responses made by individuals who are older than most participants in psychological research. This leaves open the question of whether and how these results are present in other populations. While the authors do not necessarily need to address this with more data, they should flag the possibility that age differences could play an important role in moral judgements.*

Response 5: Thank you for pointing this out. We have incorporated an additional paragraph in the “Discussion” addressing this issue. See lines 382-391.

Comment 6: *In the discussion* *authors should also add the fact that there were* *different economic incentives to participate in the study in the 2014 versus 2020 wave of data collection.*

Response 6: Thank you for pointing this out. See in the “Discussion” section lines 396-401: “Also, given logistical constraints in promptly responding to the 2020 pandemic, we could not match all test features between waves (as in the gift card given to participants in the 2014 wave). This could produce biases due to differential study enrollment in the 2014 versus 2020 wave of data collection, though at first glance the absence of a personal financial incentive in the second wave might have been expected to select for a more rather than less utilitarian cohort.”

Comment 7: *Please expand on the arguments made by competing theoretical predictions. What does it mean when Savulescu and colleagues claim “There are no egalitarian in a pandemic”? (lines 52-60)*

Response 7: Thank you for this comment; we have elaborated on the relevance of Savalescu’s claim to our research question. See lines 55-58 “In a recent article advocating for utilitarian approaches to this crisis Savulescu and colleagues [4] have claimed that “There are no egalitarians in a pandemic,” suggesting the empirical psychological claim that health crises may reduce the salience of non-utilitarian moral considerations.”

**Additional clarifications**

In addition to the above comments, we appreciate your suggestion to collect more data. However, given the unique opportunity to test participants inside and outside of a historic global pandemic, we were not able to collect further data that illuminate our key questions.

We look forward to hearing from you in due time regarding our submission and to respond to any further questions and comments you may have.
