# Supplementary material for: Reduced utilitarian willingness to violate personal rights during the COVID-19 pandemic
Source: PLoS One. 2021 Oct 22;16(10):e0259110. doi: 10.1371/journal.pone.0259110 (PMC8535394; doi:10.1371/journal.pone.0259110)
Supplement: Attachment — Submitted filename: Response to Reviewers_Sep242021.docx [file pone.0259110.s008.docx]

Dear Dr. Valerio Capraro,

Thank you for giving us the opportunity to re-submit a revised draft of the manuscript titled “No utilitarians in a pandemic? Shifts in moral reasoning during the COVID-19 global health crisis” to *PLOS ONE*. We appreciate the time and effort that you and the reviewers have dedicated to providing your valuable feedback on this manuscript. We are grateful to the reviewers for their insightful comments on the paper. We have been able to incorporate the changes to reflect the suggestions provided by reviewer #2. Below you may find a point-by-point response to reviewers’ 2 comments and suggestions.

**Comments from Reviewer #2**

Comment 1: *When you discuss the potential role of incentives in partially explaining the different results between cohorts you state: “though at first glance the absence of a personal financial incentive in the second wave might have been expected to select for a more rather than less utilitarian cohort.” Do we know how people vary in their responses to PR dilemmas based on whether or not they receive economic incentives due to their participation? While I understand the point the authors wanted to make, I do not see anything in the data or in the literature supporting this statement. Instead, I believe authors should simply acknowledge they* *do not know the magnitude or direction of the effect of financial incentives on their data.*

Response 1: Thank you for this suggestion. We agree with this suggestion and we have, accordingly, revised the paragraph and removed this conjecture (see “Discussion” section lines 407-410): “This could produce biases due to differential study enrollment in the 2014 versus 2020 wave of data collection and altered responses to the moral dilemmas tested, where the direction of such effects are unknown. ”

Comment 2: *The authors claim that “whether moral judgements are susceptible to this [age-related] effect remains unclear”. In my previous report, I pointed the authors to a recent study (Navajas et al., 2020, https://osf.io/ktv6z) that examines how demographic factors (including age) correlate with utilitarian judgements during the pandemic. [The part of my original report where I suggested authors to look at this paper was removed and did not appear in the response letter]. Discussing the relationship with those results would help better situating the findings reported in this work in the context of a fast-changing literature.*

Response 2: Thank you for this suggestion. We agree with this and we have referenced this study in the discussion section.

- We elaborated the discussion by emphasizing how demographics may affect utilitarian judgements during the pandemic (see “Discussion” section lines 382-386): “Of note, in a recent study measuring demographic factors that may affect moral judgments during the pandemic, age was negatively associated with utilitarian judgments reflecting equitable public health and positively related with judgments maximizing human life expectancy [29], indicating possibly more complex influences of age on moral considerations in the current pandemic.”
- Drawing from the Navajas 2020 paper, we additionally included a small section addressing other contextual factors such as COVID-19 severity in different countries (lines:438-440) “Other contextual factors, including regional variations in COVID-19 severity, could also affect the salience of different moral and public health considerations during this health crisis [29].

**Additional clarifications**

In addition to the above comments, we have updated the number cases in the introduction section due to COVID-19 pandemic (see line 43).

We look forward to hearing from you in due time regarding our re-submission and to respond to any further comments you may have.
